# Noninvasive Transcranial Magnetic Stimulation (TMS) in Chronic Refractory Pain: A Systematic Review

**DOI:** 10.7759/cureus.6019

**Published:** 2019-10-29

**Authors:** Pousette Hamid, Bilal Haider Malik, Mohmmed Laique Hussain

**Affiliations:** 1 Researcher, California Institute of Behavioral Neuroscience and Psychology, Fairfield, USA; 2 Internal Medicine, California Institute of Behavioral Neurosciences and Psychology, Fairfield, USA; 3 Internal Medicine, Stanley Medical College, Chennai, IND

**Keywords:** chronic pain, noninvasive management, randomized controlled trials, magnetic transcranial stimulation

## Abstract

Efficacy and tolerance of pharmacological medications in chronic pain are limited. Therefore, repetitive transcranial magnetic stimulation (rTMS) is regarded as a secure therapeutic option for pain relief, and it was proven to produce an analgesic effect. A wide variety of stimulation parameters can influence its long-lasting antalgic effect. Defining the best stimulation protocol can afford greater uniformity and consistency for considering rTMS as a promising effective tool. We aimed to systematically review and evaluate the current literature on transcranial magnetic stimulation for patients suffering from chronic pain, assess its efficacy, and estimate the best stimulation protocol. The Screened and tested electronic databases comprised PubMed, Ovid Medline, Cochrane database library, and Google scholar from the year 2000 till 2018. The keywords utilizing search terms “Transcranial magnetic stimulation”, “chronic pain”, “neuropathic pain” were used to study all possible randomized clinical trials about the impact of transcranial magnetic stimulation on long-lasting pain. All articles were judged for the possibility of prejudice using the Cochrane risk of bias tool for data extraction. Search engines produced seventy applicable results. Twelve randomized controlled clinical trials were included involving 350 patients with focal and generalized chronic pain. An existing proof showed a null response of low-frequency rTMS stimulation, rTMS delivered to the dorsolateral prefrontal cortex in chronic pain patients. However, a witnessed pain-killing response was documented when applying active high- frequency TMS on the motor cortex M1 area compared to sham. Pain relief was detected for a short time following the application of active high-frequency motor cortex stimulation in nine clinical trials, and the long-lasting analgesic effect was proved. No side effects were mentioned for the technique. Repetitive TMS can produce clinically meaningful relief from chronic pain, despite positive results, heterogeneity among all studies preclude firm conclusions regarding the optimal target stimulation site and parameters. Further studies are required to minimize bias, enhance performance, and define the best brain stimulation conditions and qualifications to maximize its potency.

## Introduction and background

*‘’ The glitch I’d like to program out of my brain, is chronic pain ... I’d like to replace my forehead with a Plexiglas window, set up a camera and film my brain and redirect it. Those areas that are generating pain *- *cool it. Those areas that are supposed to be alleviating pain* - *hello? I need you! Down-regulate pain perception circuitry, Up-regulate pain modulation circuitry*. *Now.’’ (Melanie Thernstrom, My Pain, My Brain. New York Times Magazine, 2006)*

Challenging concerns regarding chronic pain have been raised as one of the known crucial public health problems as reported by the US Institute of Medicine (IOM) that affects excess population than cardiovascular diseases and oncological disorders together. In addition to the high-cost burden of almost $560-$635 billion annually pressing on governments, it is well known that chronic pain has a negative health effect [1]. In the literature, the term chronic pain defines any pain that lasts for three months’ duration. Moreover, half of the adults admit having chronic pain, and up to 20% suffer from long-lasting relevant pain as prevalence studies indicate [2]. 

Chronic, long-lasting pain is heterogeneous. There are two types of pain: nociceptive pain, and neuropathic pain. Nociceptive pain is caused by damage or injury to tissues, while neuropathic pain is due to preceding damage directed towards pain neurons leading to the spontaneous firing of action potential either originating from the central or peripheral nervous system in spite of the disappearance of any noxious stimulus. Neuropathic pain is difficult to alleviate; patients may not respond adequately to pharmacological therapies, and other therapeutic alternative options to renovate neurons are still in the trial phase [3].

In these settings, non‐pharmacological interventions are highly advocated. Cortical stimulation has surfaced as a promising, interesting, and effective modality as a novel approach to control chronic pain. Modification of neuronal action potential excitability in the neural circuits concerned with pain processing signaling, by either inhibition or interruption of these thalamic pain signals and other hyperactive localization pain network, is the assumed mechanism of action. Recently, there has been a considerable growing interest in cortical stimulation which is suggested to interfere with the neural connections responsible for pain modulation [4].

Transcranial magnetic stimulation has been discovered to create an analgesic impact by stimulating the primary motor cortex (M1). It was found that applying "high-frequency" rTMS (e.g., stimulation frequency ranging from 5 to 20Hz) to the precentral gyrus (e.g., M1 region), is responsible for attaining a pain relief response through stimulation of enormous distant cortical areas responsible for pain modulation. There is considerable proof to acknowledge high pain control when using high-frequency rTMS of the primary motor cortex (M1) contralateral to the pain localization with level A definite effectiveness, according to Lefaucheur and his colleagues [5].

Researchers have studied the duration of pain relief after TMS. They mentioned that in the case of repeated stimulation technique, analgesia could last for weeks beyond the stimulation, which could be attributed to long-term synaptic plasticity, and wide-spread effects reaching remote brain areas other than the cortex [6]. TMS application is done utilizing a figure-of-eight shaped coil stimulating the scalp, which it is supposed that its effect can spread to diverse areas of pain network experience. A pain scientist confirmed at Stanford University, California, USA, that pain is not one unit; on the contrary, it has multiple dimensions. Being heterogeneous in its sources, it also varies in its qualities and specifications. Sensing somatic pain differs from the associated awful emotional suffering [7]. TMS seems to affect both aspects. Recovery of the pain could be due to modification of the downward thalamic pathway from the brainstem to the spinal cord, primary and secondary somatosensory cortex, or involving pain modulation diencephalic system. On the other hand, any control over the emotional elements of pain is probably due to its impact on the limbic system connections (the anterior cingulate and insular cortices) [8,9]. According to guidelines, the motor cortex is the preferred localization in terms of targeting TMS for the management of pain. Novel targets have been explored in few studies like the dorsolateral prefrontal cortex (DLPFC) in chronic depression patients; however, it showed poor response in pain relief in some studies and limited beneficial analgesic effects in others like clinical trials in migraine [10,11].

Repetitive TMS (rTMS) has been identified as repeated rapid successive stimulation pulses to be supplied in one session. Nowadays, it is considered as the favorite stimulation technique. The application technique differs from one study to the other concerning rTMS device used, the shape of the coil, assigned location, frequency and intensity of stimulation, trains number and duration, the total number of sessions required, and total pulses applied. The precise best method may differ from one patient to another [10]. It is evident that variable outcomes can happen according to the target area stimulated and which frequency has been applied and selected.

Interestingly, inhibition of the neuronal function was noticed upon delivery of low frequencies (≤1 Hz), whereas cortical excitation can happen in case of high frequencies stimulation (≥5 Hz). Left prefrontal cortical stimulation was known to be connected with antidepressant and mood stabilization impact, whereas, meaningful analgesic effects can be demonstrated upon repeated stimulation of the contralateral primary motor cortex (M1) particularly. A current meta‐analysis reported that rTMS could be extremely efficient in neuropathic pain management, especially conditions with a central origin rather than being peripheral [12].

The duration of pain relief varies among different studies, most of them declared short term improvement in pain sensation after a single session of rTMS, however, this relief can be extended by applying repeated sessions of TMS reaching from three weeks to few months after the end of sessions [13]. The pain level was quantitatively measured at baseline, after first, during, and after completion of the sessions. Long-term maintenance rTMS protocol can be of therapeutic benefit in the management of patients with chronic refractory pain, although the exact pathophysiology is not fully understood.

Determining maintenance therapy regimes, based on the absolute paradigmatic application model has to be recognized in larger trials. This systematic review aims to evaluate the repetitive transcranial magnetic stimulation analgesic influence on chronic refractory pain, particularly central neuropathic pain in adults in regards to variable stimulation and localization parameters.

## Review

Methodology

Recommendations of the preferred reporting items for systematic reviews and meta-analyses (PRISMA) statement have been accomplished [14]. Medline/ PubMed, Web of Science, Cochrane database, and Google Scholar were searched to identify relevant studies.

Criteria for Incorporation

We included randomized controlled clinical trials, parallel or cross-over studies, of repetitive TMS irrespective of the protocol used, published from 2000 to 2018. Sham-controlled, peer-reviewed studies on adults > 18 years old (diagnosed with chronic neuropathic pain), published in English, with a clear primary outcome of pain intensity and quantitative measurement either by visual analog scales (VAS) or pain measurement rating scales were included.

Exclusion Criteria

All clinical trials were excluded if they were: non-randomized, not in English, not sham-controlled, if the pain was not confined to chronic pain, or involvement of acute pain, or pain as an outcome was not properly estimated.

Cochrane risk-of-bias tool for randomized controlled trials was used to assess the possibility of bias in the included studies [15]. The criterion evaluated for parallel/cross-over models of the trials (using low/high/unclear judgments) were: appropriate generation of sequence, adequate concealment of allocations, proper blinding of evaluators and participants, sufficient evaluation of incomplete results, confirmation that they were devoid of selective reporting of results, and lacking of other bias.

Primary Outcome Measures

To assess the change in pain intensity levels, the included articles used measurements like visual analog scales (VAS), Leeds assessment of neuropathic symptoms and signs (LANSS) scale, or other quantitative scales before, during, and following 14 days after last application. 

Data Extraction and Study Variables

All the following variables were investigated, and all details concerning the clinical studies were mentioned clearly: the country of origin, risk-of-bias assessment of the studies, designs, population incorporated, estimated size of the sample, for both active and control groups. Different stimulation parameters, with precise sham justification credibility mentioning how closely it is distinguishable from active stimulation. An uncertain judgment was reached in case the researcher did not properly describe the sham situation. Clear and adequate pain scoring at all follow-up points were noted, keeping in consideration that adverse effects had to be ruled out. Finally, disclosure of conflict of interest was mentioned. 

Results

**Figure 1:**
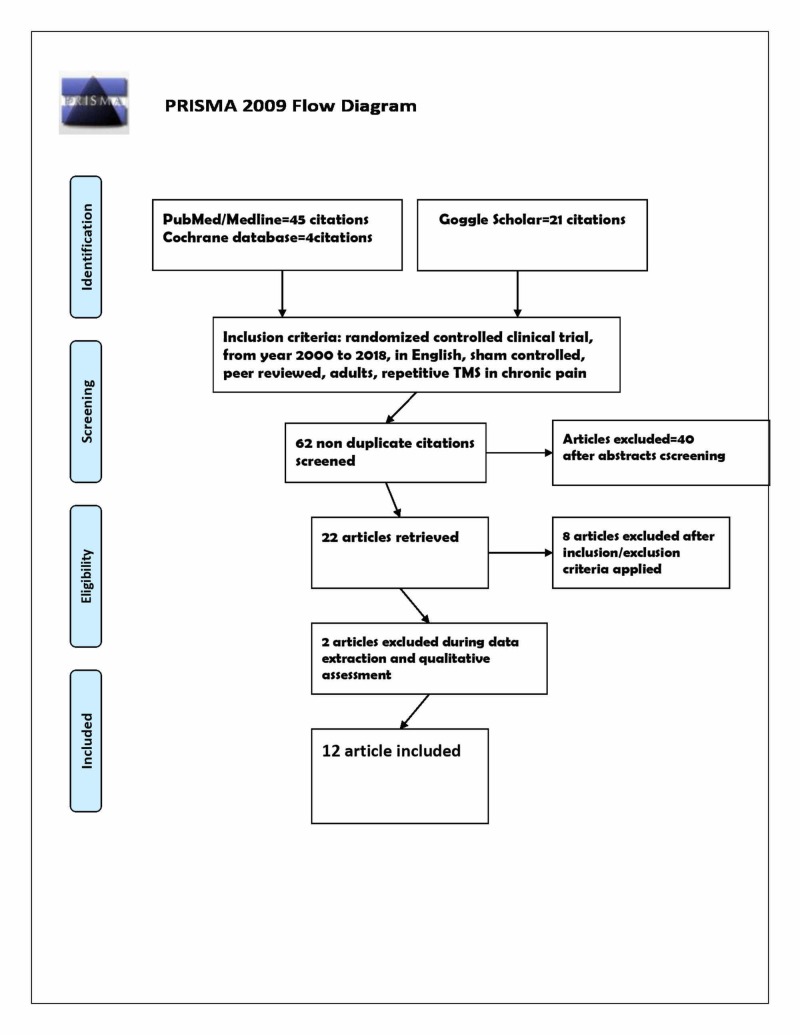
PRISMA flow diagram of Repetitive TMS in chronic pain

Seventy studies were screened from the titles and abstracts using a predefined search strategy initially, with the help of previously mentioned MeSH and regular keywords. Eight were excluded as duplication. Forty were excluded for being non-randomized studies, two were excluded for lacking extractable data, and eight were excluded because no sham control was mentioned. Twelve randomized clinical studies were included according to inclusion and exclusion criteria. The selection process PRISMA flow diagram is described in Figure 1, and the features of the included research are outlined in Table 1.

**Table 1:** Characteristics of included randomized controlled trials Randomized controlled study (RCT) Visual Analogue Scale (VAS) Dorsolateral prefrontal cortex (DLPFC) Somatosensory cortex (SII) Motor cortex (MC)

Author	Year	Country	Sample	TMS freq	TMS site	Conclusion
1- Khedr et al. [16]	2005	Egypt Cross-over randomized controlled trial ( RCT)	48	20HZ	Motor cortex (MC)	Significant reduction of pain immediately and maintained for 1 month using Visual Analog Scale (VAS) for pain
2- Passard et al. [9]	2007	France Parallel RCT	30	10 HZ	Left MC	minimal reduction of pain long lasting for 2 weeks
3- Carretero et al. [17]	2009	Spain Parallel RCT	26	1 HZ	Dorsolateral prefrontal cortex (DLPFC)	VAS reduction of pain
4- Borckardt et al. [11]	2011	USA Cross-over	20	10HZ	DLPFC	Significant VAS reduction of pain, immediate and short-termed
5- Fregni et al [18]	2011	USA Parallel RCT	17	1 HZ	Somatosensory cortex (SII)	VAS reduction in pain
6- Ahmed et al. [19]	2011	Egypt Cross-over	27	20 HZ	Motor area1 (M1)	Significant VAS reduction in pain long lasting for 1 month
7- Andre-Obadia et al. [20]	2011	France Cross-over	45	20 HZ	M1	Significant VAS reduction in pain
8- Avery et al. [21]	2013	USA Parallel RCT	19	10 HZ	left dorsolateral prefrontal (LDLPFC)	Unclear benefit
9- Hosomi et al. [22]	2013	Japan Cross-over	70	5 HZ	M1	Significant immediate VAS reduction in pain
10- Conforto et al. [23]	2013	Brazil Parallel	18	High frequency	DLPFC	Absence of significant benefit in active group
11- Shimizu et al. [24]	2017	Japan Cross-over	18	5 HZ	M1	VAS reduction in pain (short term)
12- Andre-Obadia et al. [25]	2018	France Cross-over	12	20 HZ	M1	Significant pain reduction

Raw data of 350 patients were extracted from 12 clinical trials (five parallel, seven cross-over) selected from 70 articles. The mean duration time of neuropathic pain was more than three months. Studies included focal neuropathic pain, generalized pain like fibromyalgia. Our results demonstrated a statistically significant (P < .001) analgesic impact with pain improvement according to the mean percent reduction in pain visual analog scale (VAS) score with a higher decrease of rTMS VAS compared to sham. The applicable rTMS frequencies were (1 HZ, 5 HZ, 10 HZ, 20 HZ). Eight clinical trials used high-frequency TMS; four used low frequencies. The target site for stimulation was M1 contralateral to the painful site in seven studies, DLPFC in four studies, SII in only one clinical trial. The number of sessions of rTMS ranged from a single session to five successive or 10. Not all studies clearly specify sham blinding, whether they used inert or active sham stimulation. Pain scores were compared for the experimental group to the sham group at baseline, during the sessions, and two to eight weeks post-stimulation. 

Follow up period after the stimulation varies from a study to another. Long-lasting analgesic effect was detected in three clinical trials (Passard et al., Khedr et al., and Ahmed et al.) for 2-4 weeks, in contrast to short term analgesic effect after stimulation in nine studies [9,16,19].

Quality Appraisal of the Trials

The risk of bias differs from one trial to another regarding the assessment criteria. It is fundamental to state that whenever the randomization was clear and specific, the more any study has a low risk of bias. If the description of randomization is not clearly defined, studies are with an unclear risk of bias. The study was said to be of a high risk of bias if randomization has not been precisely achieved, for example in Khedr et al. and Ahmed et al., where the patients were randomized based on the day of the week on which they were recruited [16,19]. We believe that all clinical trials attempted to blind respondents. See Table 2 for clarification of the risk of bias assessment across the research.

**Table 2:** Quality assessment of RCT using the Cochrane risk-of-bias tool

RCT	Selection bias	Reporting bias	Performance bias	Detection bias	Attrition bias
1- Khedr et al. [16]	Low risk	Low risk	High risk	Low risk	Unclear
2- Passard et al. [9]	Low risk	Low risk	low risk	Unclear	Low
3- Carretero et al. [17]	Unclear	Low	High	Low	Low
4- Borckardt et al. [11]	Low	Low	Low	Low	Low
5- Fregni et al. [18]	Low risk	High	Low	Unclear	Unclear
6- Ahmed et al. [19]	High	Low	Low	High	Low
7- Andre-Obadia et al. [20]	Low	Low	Low	Unclear	Low
8- Avery et al. [21]	Low	Low	Low	Low	Low
9- Hosomi et al. [22]	Low risk	High risk	Low risk	Low risk	Low risk
10- Conforto et al. [23]	High	Low	Low	High	Low
11- Shimizu et al. [24]	Low	Unclear	Low	Low	Low
12- Andre-Obadia et al. [25]	Low	Unclear	High risk	Low risk	Low

Results clearly defined the analgesic effect of TMS when used repetitively, high frequency, on the M1 area. There is substantial uncertainty about the possible benefits of low-frequency rTMS and rTMS applied to the prefrontal areas of the brain. A cumulative analgesic impact was demonstrated in the case of multiple sessions of repetitive TMS and an increased amount of pulses per session. Noninvasive brain stimulation and sham stimulation appear to be associated with a negligible adverse effect.

Discussion

This review focused on evaluating the best available evidence of repetitive TMS in amelioration of chronic refractory neuropathic pain. The success of pain modulation due to TMS repetitive technique is, in fact, due to parameters of stimulation. These include primarily target brain area, which varies among distinct studies. Frequency applied in different protocols as well as pulses. Interestingly, the outcome is dependent on the total performed number of sessions.

 Due to the stimulation of M1 with elevated frequencies (about 5 Hz) (proof level A) in neuropathic pain, a definite analgesic impact was noted, and its use is suggested for the treatment of pain illnesses [5]. Many authors confirmed and verified that high-frequency motor cortex rTMS decreases chronic pain [26]. Nevertheless, researchers have been investigating the most beneficial M1 region that should be targeted, whether somatotopic facial or hand region depiction. The results of various research showed that stimulation of the hand region in most research resulted in a significant reduction in pain compared to the face region [27,28]. Based on Migita and his colleagues, repetitive TMS over the M1 area in patients suffering from central pain has been associated with 30% pain relief [29].

DLPFC area stimulation can lead to pain reduction in some chronic pain conditions present within depressive symptoms and fibromyalgia [30]. Remarkably, in three patients having neuropathic pain, the produced analgesic impact was independent of mood scores, as mentioned by Borckardt and his colleagues [11]. Nevertheless, some other researchers found the absence of any significant analgesic effect upon stimulation of the DLPFC area [30]. De Oliveira et al. reported the same results of the lack of any antinociceptive effect of stimulation of rTMS in the DLPFC area [31]. 

Stimulation of the S2 region was hypothesized to generate analgesic impacts owing to the closeness of this region and greater anatomical relationships in pain perception with strategic fields, known as the 'pain matrix' [32].

The follow-up period for the intervention is not the same in all included studies; some showed durable analgesic effects which are maintained for three weeks after stimulation in focal neuropathic pain as in study by Khedr et al., or long-lasting effect in generalized pain as in a study done by Passard et al. [9,16]. The impacts of rTMS on affective pain were longer lasting than on sensory pain, indicating differential impacts on brain structures engaged in pain perception [9]. Most of the included studies are inconsistent with other researchers who admit delayed analgesic effects for repeated sessions of rTMS after five days of stimulation [12,33]. Kobayashi, in his study, mentioned that in 61.1 percent of patients with central pain at the 12th week, rTMS (10 trains of 10-second 5 Hz-rTMS) of M1, sustained once a week, was efficient. He confirmed a sustainable, long-lasting antalgic effect in six patients following one year of rTMS continuation [34]. This effect seems to be linked to several pulses per session; 2000 in Khedr et al., 1000 for Lefaucheur et al., 400 for Topper et al. [5,16,33]. 

The mechanism of action of rTMS is still unclear. However, intracortical facilitation (ICF) in responders (30% decrease in pain after rTMS) was smaller at baseline, and it increased considerably after rTMS, suggesting that its pain modulation could be associated with restoring abnormal cortical excitability in chronic primary pain [34]. Lack of homogeneity was evident among studies, pain disorders were studied with distinct pathophysiological processes and aetiologies, so it was somewhat hard to compare the outcomes of these studies. In most of the involved trials, there was an abundant risk of bias in variable ways. Lack of clarity of randomization is present in some of them. We found considerable variation in the measurement of quantitative pain scores in all studies with variable pain scales used, adding more to a load of bias. Thus, making the interpretation quite complex.

Limitations of the Review

Heterogeneity of different studies, incomplete analysis of the full degree of pain relief, variations in the target stimulation site, and inconsistency in stimulation parameters were the most significant limitations.

## Conclusions

This review evaluated the pain reduction effect of repetitive transcranial magnetic stimulation in chronic pain. Although TMS is a safe, promising technique to reduce long-lasting refractory pain, still the evidence is hampered and influenced by multifactorial stimulation parameters. Additional research efforts are needed to highlight the best optimal stimulation protocol and to standardize all parameters to promote the long-term efficacy of rTMS as a noninvasive alternative in the management of chronic refractory pain.
